# Entwicklung der Zahn- und Mundgesundheit in Deutschland von 1997 bis 2014

**DOI:** 10.1007/s00103-021-03345-6

**Published:** 2021-06-18

**Authors:** Thomas Kocher, Birte Holtfreter, Vinay Pitchika, Kathrin Kuhr, Rainer A. Jordan

**Affiliations:** 1 Poliklinik für Zahnerhaltung, Parodontologie, Endodontologie, Kinderzahnheilkunde und Präventive Zahnheilkunde, Universitätsmedizin Greifswald, Fleischmannstraße 42, 17475 Greifswald, Deutschland; 2 Institut der Deutschen Zahnärzte (IDZ), Köln, Deutschland

**Keywords:** Epidemiologie, Trend, Karies, Parodontitis, Zahnzahl, Epidemiology, Trend, Caries, Periodontitis, Number of teeth

## Abstract

**Hintergrund:**

Zur Veränderung der oralen Krankheitslast in der deutschen Allgemeinbevölkerung über die letzten 20 Jahre gibt es bisher keine umfassende Darstellung.

**Fragestellung:**

Wie haben sich die Prävalenzen der Karies, der Parodontitis und des Zahnverlustes und deren Determinanten von 1997 bis 2014 in Deutschland verändert?

**Material und Methoden:**

Ausgewertet wurden Daten von 35- bis 44- und 65- bis 74-Jährigen der Deutschen Mundgesundheitsstudien (DMS) III, IV und V sowie von 25- bis 74-Jährigen der Studies of Health in Pomerania (SHIP‑0 und SHIP-Trend-0). Der Decayed, Missing, Filled Teeth Index (DMFT), die Anzahl füllungsfreier Zähne, der Community Periodontal Index (CPI) als auch Daten zur Zahnzahl und Zahnlosigkeit wurden herangezogen.

**Ergebnisse:**

In beiden Studien waren bzgl. der Determinanten eine Zunahme der Probanden mit hoher Schulbildung, eine geringfügige Abnahme der Raucher sowie eine Verbesserung der Mundhygiene zu beobachten. Bei den 35- bis 44-Jährigen stieg die Anzahl gesunder Zähne von 11,9 in DMS III auf 16,8 in DMS V, während bei den Senioren die Anzahl gesunder Zähne um 5,9 anstieg. In SHIP wurde ein ähnlicher Trend beobachtet. Die Prävalenz des höchsten CPI-Grades 4 fiel in den DMS bei den 35- bis 44-Jährigen von 9,3 % auf 3,5 %; bei den Senioren lag der Wert 2014 wieder auf dem Niveau von 1997 (10,5 % und 9,8 %). Parallel dazu hat sich der Anteil der zahnlosen Senioren in beiden Studien halbiert. Die Zahnzahl nahm über alle Altersklassen hinweg zu.

**Diskussion:**

DMS und SHIP zeigten über die Jahre konsistent eine Zunahme gesunder, füllungsfreier Zähne, eine geringfügige Reduktion der Probanden mit CPI-Grad 4 sowie mehr Zahnerhalt und weniger Zahnlosigkeit. Bedingt durch den höheren Zahnerhalt und die Alterung der Gesellschaft ist in Zukunft mit einer erhöhten parodontalen Behandlungslast zu rechnen.

**Zusatzmaterial online:**

Zusätzliche Informationen sind in der Online-Version dieses Artikels (10.1007/s00103-021-03345-6) enthalten.

## Abstract (en)

**Background:**

Currently, there is no comprehensive presentation of trends in oral diseases in the German general population over the last 20 years.

**Objectives:**

How did prevalences of caries, periodontitis, and tooth loss and their determinants change in Germany between 1997 and 2014?

**Materials and methods:**

We analysed data from 35- to 44-year-olds and 65- to 74-year-olds from the German Oral Health Studies (“Deutsche Mundgesundheitsstudien” [DMS]) III to V and of 25- to 74-year-olds from the Studies of Health in Pomerania (SHIP‑0 and SHIP-Trend-0). The decayed, missing, filled teeth index (DMFT), the number of sound teeth, the community periodontal index (CPI), and data on tooth count and edentulism were analysed.

**Results:**

Regarding determinants, an increase in subjects with high school education, a slight decrease in smokers, and an increase in better oral hygiene patterns was observed in both studies. In 35- to 44-year-olds, the number of sound teeth increased from 11.9 in DMS III to 16.8 in DMS V, while in 65- to 74-year-olds the number of sound teeth increased by 5.9. A similar trend was observed in SHIP. In DMS, the prevalence of the highest CPI score of 4 decreased from 9.3% to 3.5% in 35- to 44-year-olds; in 65- to 74-year-olds, the 2014 prevalence was at the same level as in 1997 (10.5% and 9.8%). In parallel, the percentage of edentulous 65- to 74-year-olds halved in both studies. The number of teeth increased across all age strata.

**Conclusions:**

DMS and SHIP consistently showed an increase in the number of healthy teeth free of fillings, a slight reduction of subjects with a CPI score of 4, more tooth retention, and less edentulism. Because of more tooth retention and current demographic changes, higher periodontal treatment needs might be expected for the future.

## Einleitung

Karies und Parodontitis haben einen großen Anteil am Krankheitsgeschehen in der Mundhöhle. Karies ist ein bakteriell bedingter Prozess, bei dem die Zahnhartsubstanz durch Säure langsam abgebaut wird und schließlich eine Aushöhlung (Kavität) im Zahn entsteht, die Schmerzen verursachen kann. Ist zu viel Zahnhartsubstanz verloren gegangen oder kann der Schmerz nicht beseitigt werden, so muss der Zahn extrahiert werden. Parodontitis ist eine bakterielle Entzündung des Zahnhalteapparats, die schleichend beginnt, vom Patienten oft unbemerkt bleibt und zum Abbau von Knochen führt. Bleibt sie lange Zeit unbehandelt, so kann sie zu Lockerung und letztendlich zu Zahnverlust führen.

Wegen Karies und Parodontitis werden über alle Altersklassen hinweg in Deutschland ca. 60–70 % aller Zähne extrahiert, die restlichen 30–40 % werden aus prothetischen, kieferorthopädischen Planungsgründen oder wegen eines Traumas extrahiert [1]. Karies kann schon im Milchgebiss zu Extraktionen führen und erreicht die größte Bedeutung bei den 30- bis 40-Jährigen, während parodontal bedingte Zahnextraktionen bei den 50- bis 60-Jährigen ihren Höhepunkt erreichen [1]. Für die meisten Patienten ist ein Zahnverlust ein einschneidendes Ereignis, insbesondere wenn die extrahierten Zähne durch eine herausnehmbare Prothese ersetzt werden müssen. Zahnverlust kann zu einer eingeschränkten Kaufunktion und ästhetischen und funktionellen Einschränkungen führen, wodurch sich die mundgesundheitsbezogene Lebensqualität erheblich verschlechtert [2].

Metaanalysen der Global-Burden-of-Disease-Studie des US-amerikanischen Institute for Health Metrics and Evaluation (IHME) aus den Jahren 2014 und 2015, die sich auf Daten aus allen Kontinenten stützten, konnten zeigen, dass sowohl die Prävalenz von starkem Zahnverlust (weniger als 10 Zähne im Mund) als auch dessen Inzidenz von 1990 bis 2010 um 50 % abnahm, während sich die weltweiten Prävalenzen und Inzidenzen von manifester Karies und schwerer Parodontitis nicht veränderten [3–5]. Allerdings ist bekannt, dass die Kariesprävalenz von Kindern und Jugendlichen in den industrialisierten Ländern seit den 1970er-Jahren sehr stark gesunken ist und dass der Kariesrückgang in Deutschland im Vergleich zu den anderen europäischen Ländern und den USA erst verspätet einsetzte. In schwedischen Querschnittsuntersuchungen in Jönköping konnte gezeigt werden, dass sich der Kariesrückgang auch bei den 20- bis 80-Jährigen auswirkte und zu einem geringeren Zahnverlust über alle Altersstrata führte [6]. Hingegen ist noch immer unklar, ob und wie stark sich die Prävalenz von moderaten bis schweren Parodontalerkrankungen im Laufe der letzten Dekaden in der westlichen Welt verändert hat. Ein Problem der Erhebung der Parodontitisprävalenz ist die zeitaufwendige Untersuchung. Viele Studien nehmen nur einen Teilbefund auf oder untersuchen lediglich Indexzähne, weshalb es nur wenig verlässliche Daten gibt [7].

Durch repräsentative, bevölkerungsweite Gesundheitssurveys können Prävalenzen von Krankheiten und deren Determinanten erfasst und damit vergangene Entwicklungen analysiert und zukünftige Trends eventuell extrapoliert werden. Gesundheitssurveys sind eine Voraussetzung, um Strukturen eines Gesundheitssystems nachhaltig und effektiv zu verändern bzw. zu verbessern. Neben Krankheiten können sich auch im Laufe der Jahre die Prävalenzen der Determinanten verändern. Zum Beispiel rauchten durch Maßnahmen des Gesetzgebers im Lauf der letzten Dekaden immer weniger Männer in Deutschland, was sich in der geringeren Anzahl von Lungenkrebserkrankten widerspiegelt. Nur durch wiederholte Querschnittsuntersuchungen können Prävalenzveränderungen sowohl einer Krankheit als auch ihrer Determinanten erfasst werden. Wenn sowohl zu den Krankheits- als auch zu den Risikofaktoren Prävalenzangaben vorliegen, kann ermittelt werden, ob sich durch die Prävalenzveränderung eines Risikofaktors auch die Krankheitslast verändert hat.

Die Epidemiologie oraler Erkrankungen stellt 2 grundsätzliche Fragen [8]: 1) Wie hoch ist die Prävalenz in der Bevölkerung und 2) was sind die wichtigsten Risikofaktoren? Vom Public-Health-Standpunkt aus ist es wichtig, diese Fragen zu beantworten. Zum einen muss entschieden werden, in welchem Umfang Ressourcen nötig sind, um die Prävalenz in der Bevölkerung zu reduzieren, zum anderen müssen die Risikofaktoren identifiziert werden, die wichtig sind, um erfolgreiche Interventionsstrategien auf Bevölkerungsebene zu erarbeiten [9]. Die aussagekräftigsten Indikatoren für Mundgesundheit sind Karies und Parodontitis und als deren Folge der Zahnverlust. In dieser Arbeit sollen die Veränderungen der Prävalenzen von Karies, Parodontitis und Zahnverlust im Zeitraum 1997 bis 2014 anhand der Deutschen Mundgesundheitsstudien (DMS) des Instituts der Deutschen Zahnärzte (IDZ) und der Study of Health in Pomerania (SHIP) der Universitätsmedizin Greifswald dargestellt werden und mögliche Gründe für die Veränderungen des Zahnverlustes diskutiert werden.

## Methoden

### Deutsche Mundgesundheitsstudien (DMS)

DMS sind multizentrische, landesweite Querschnittsstudien der Bevölkerung Deutschlands [8, 10, 11]. Insgesamt wurden 90 Untersuchungsgemeinden (60 in den alten und 30 in den neuen Bundesländern) anhand einer mehrstufig geschichteten Zufallsstichprobe gezogen, die nach Regionen und Gebieten der Urbanisierung geschichtet war. Über die jeweiligen Einwohnermeldeämter wurden anschließend die Studienteilnehmer (Kinder, 35- bis 44-jährige Erwachsene, 65- bis 74-jährige Senioren, in DMS V auch 75+-jährige Senioren) als geschichtete Stichprobe gezogen. Die Untersuchungen für DMS III, IV und V erfolgten jeweils 1997, 2005 und 2014 mit einer Response von jeweils 63 %, 63 % und 50 %. Für die vorliegende Studie wurden Daten von 35- bis 44-jährigen Erwachsenen und von 65- bis 74-jährigen Senioren ausgewertet, da nur für diese beiden Altersgruppen durchgehend Daten zur Prävalenz von Karies, Parodontitis und Zahnzahl vorlagen. Für die Analysen wurden somit 2022 Teilnehmer der DMS III, 1965 Teilnehmer der DMS IV und 2008 Teilnehmer der DMS V eingeschlossen.

### Studies of Health in Pomerania (SHIP)

SHIP sind regionale Studien in Vorpommern. Für SHIP‑0 wurde eine repräsentative Stichprobe von 7006 Personen mit einem Alter von 20 bis 79 Jahren in einem zweistufigen Clusterstichprobenverfahren gezogen [12, 13]. Die Auswahl erfolgte proportional zur Bevölkerung der Gemeinden, stratifiziert nach Alter und Geschlecht. Die Erstuntersuchung SHIP‑0 wurde von 1997 bis 2001 durchgeführt und umfasste 4308 Teilnehmer (Response 69 %). Für SHIP-Trend‑0 [14] wurde in demselben Gebiet eine weitere stratifizierte Zufallsstichprobe von 10.000 Erwachsenen im Alter von 20 bis 79 Jahren gezogen. Zwischen 2008 und 2012 wurden 4420 Teilnehmer untersucht (Response 50 %). In dieser Publikation beschränken wir uns auf die 25- bis 74-Jährigen, um eine Vergleichbarkeit der Daten zu den in der DMS selektierten Altersgruppen zu ermöglichen.

### Karies

Die Karieserfahrung wurde in DMS und SHIP mittels des DMFT(Decayed, Filled, Missing Teeth)-Index erfasst. In DMS wurden alle 28 Zähne (ohne Weisheitszähne) untersucht; in SHIP erfolgte eine halbseitige Befundung auf der rechten oder linken Kieferseite (maximal 14 Zähne, ohne Weisheitszähne). Die folgenden Befunde wurden registriert: i) „sound tooth“ (ST) bezeichnet einen Zahn ohne Kavität oder Restauration, ii) „decayed tooth“ (DT) bezeichnet einen Zahn, bei dem eine Kavität deutlich zu sehen oder mit einer stumpfen Sonde zu ertasten ist, iii) „filled tooth“ (FT) bezeichnet einen Zahn mit einer oder mehreren dauerhaften Restaurationen und ohne Kavitation, iv) „missing tooth“ (MT) bezeichnet einen Zahn, der aus irgendeinem Grund verloren gegangen ist oder extrahiert wurde.

### Zahnzahl

Die Zahnzahl ergibt sich als Anzahl der natürlichen Zähne (inkl. Wurzelreste) ohne Berücksichtigung der Weisheitszähne und variiert somit zwischen 0 und 28. Waren alle 28 Zähne extrahiert, so wurde der Proband als „zahnlos“ bezeichnet. Dies gilt auch bei Vorhandensein von dentalen Implantaten: Sie stellen einen Zahnersatz dar, weil ihnen ein natürlicher Zahnverlust vorausging.

### Parodontitis

Zur Erfassung der Parodontitis wurden die Sondierungstiefen und in SHIP zusätzlich die Attachmentverluste (Verlust des parodontalen Halteapparates durch parodontale Entzündungen) erhoben. Die Sondierungstiefe entspricht der Strecke vom Zahnfleischrand bis zum Boden der parodontalen Tasche; sie ist ein Maß für die aktuelle Erkrankung. Der Attachmentverlust entspricht der Strecke von der Schmelz-Zement-Grenze bis zum Boden der parodontalen Tasche; er ist ein Maß für die über die Lebenszeit akkumulierte Erkrankung. Eine detaillierte Darstellung der Messmethodik kann dem Onlinematerial entnommen werden. Es wurden die mittlere Sondierungstiefe und der mittlere Attachmentverlust bestimmt.

Parodontal erkrankte Probanden wurden entsprechend des Community Periodontal Index (CPI) klassifiziert: Ein CPI-Grad 3 zeigt eine moderate Parodontitis an und bedeutet, dass der Proband an mindestens einer Zahnfläche eine Sondierungstiefe von 3,5–5,5 mm aufwies. Bei einem CPI-Grad 4 lag eine schwere Parodontitis vor; es wurde an mindestens einer Fläche eine Sondierungstiefe > 5,5 mm nachgewiesen. Parodontale Gesundheit entspricht einem CPI-Grad von 0–2.

Um über die einzelnen DMS eine Vergleichbarkeit der abgeleiteten CPI-Prävalenzen zu erreichen, erfolgte eine Einschränkung auf die in DMS III, IV und V überlappend befundeten Flächen und Zähne. Somit wurden die CPI-Grade basierend auf den Sondierungstiefenwerten der mesiobukkalen (vorne und zur Wange hin gelegenen) und mittbukkalen (mittig zur Wange gelegenen) Flächen der Zähne 17, 16, 11, 44, 46, 47 ermittelt.

### Interview

In SHIP und in DMS wurde auch ein zahn- und allgemeinmedizinisches Interview durchgeführt sowie demografische Determinanten erfasst: Geschlecht, Alter, Schulbildung (< 10/10/> 10 Jahre), Rauchen (Nie-Raucher/Ex-Raucher/Raucher), Verwendung einer elektrischen Zahnbürste (Ja/Nein), Interdentalhygiene (Gebrauch von Zahnseide, Zahnhölzchen/-stocher/-stäbchen oder Zahnzwischenraumbürstchen; Ja/Nein), Verwendung von Mundwasser (Ja/Nein), Zahnputzhäufigkeit (≥ 2-mal täglich, < 2-mal täglich), durchgeführte Parodontalbehandlung (DMS und SHIP: innerhalb der letzten 5 Jahre), Diabetes mellitus (DMS: mit und ohne Insulinbehandlung; SHIP: Selbstangabe einer ärztlichen Diagnose oder Einnahme antidiabetischer Medikamente), Kontrollbesuche beim Zahnarzt („Gehen Sie zum Zahnarzt nur, wenn Sie Schmerzen oder Beschwerden haben? Oder gehen Sie regelmäßig oder manchmal auch zur Kontrolle?“; Kontrolle vs. Schmerzen/Probleme/nie), Zahnarztbesuch innerhalb der letzten 12 Monate (Ja/Nein), Selbstwirksamkeit (DMS: „Wie viel kann man selbst tun, um die Gesundheit seiner Zähne zu erhalten oder zu verbessern?“; SHIP: „Wie viel kann man selbst tun, um die Gesundheit seiner Zähne zu erhalten oder zu fördern?“; sehr viel/viel/einiges/wenig/nichts). Für DMS wurde zusätzlich der Wohnort (alte/neue Bundesländer) erfasst. In SHIP wurden zusätzlich Angaben zu Hämoglobin A1c (HbA1c), zur physischen Aktivität (≥ 1 h pro Woche im Sommer oder Winter) und zum Einkommen (in Euro; als Äquivalent für 2012 nach Korrektur für die jährlichen Inflationsraten) erhoben.

### Statistische Analysen

Die deskriptive Darstellung der kontinuierlichen Variablen erfolgte als Mittelwert, teils in Kombination mit der Standardabweichung. Für kategoriale Variablen wurden die absoluten und relativen Häufigkeiten angegeben.

## Ergebnisse

### Veränderungen der Determinanten für Zahn- und Mundgesundheit

In DMS nahm zwischen 1997 und 2014 die Anzahl der Probanden mit einer > 10-jährigen Schulbildung sowohl bei den 35- bis 44-Jährigen als auch den 65- bis 74-jährigen Senioren um 14 % und in SHIP über alle Alterstraten hinweg um 9 % zu (Tab. 1). In beiden Studien nahm der Anteil der gegenwärtigen Raucher geringfügig ab: in DMS bei den 35- bis 44-Jährigen von 37,7 % auf 28,5 %; bei den DMS-Senioren und SHIP-Probanden war die Abnahme auf wenige Prozente beschränkt. In der DMS stieg die Anzahl der Probanden, die eine elektrische Zahnbürste benutzten, in beiden Altersgruppen um etwa 30 %. Für Interdentalhygienemittel wurde in der DMS eine Zunahme von 32 % bzw. 42 % verzeichnet. Zudem stieg in DMS die Anzahl der Probanden, die aus Kontrollgründen den Zahnarzt aufsuchen, um 7 % auf 76,7 % bei den 35- bis 44-Jährigen bzw. um 34 % auf 91,4 % bei den 65- bis 74-Jährigen.

**Table 1:** 

	DMS III (1997)				DMS V (2014)				SHIP‑0 (1997–2001)		SHIP-Trend‑0 (2008–2012)	
	*N*	35- bis 44-Jährige	*N*	65- bis 74-Jährige	*N*	35- bis 44-Jährige	*N*	65- bis 74-Jährige	*N*	25- bis 74-Jährige	*N*	25- bis 74-Jährige
Alter (Jahre)	655	39,5 ± 2,8	1367	69,0 ± 2,8	966	39,8 ± 2,9	1041	69,4 ± 3,0	3739	49,6 ± 13,9	3971	50,8 ± 13,5
Weibliches Geschlecht	655	345 (52,7)	1367	756 (55,3)	966	513 (53,1)	1042	552 (53,0)	3739	1914 (51,2)	3971	2067 (52,1)
*Schulbildung*												
< 10 Jahre	649	184 (28,3)	1360	1030 (75,7)	964	160 (16,6)	1006	480 (47,7)	3717	1436 (38,6)	3959	804 (20,3)
10 Jahre	–	279 (43,0)	–	175 (12,9)	–	391 (40,6)	–	266 (26,5)	–	1693 (45,6)	–	2159 (54,5)
> 10 Jahre	–	186 (28,7)	–	155 (11,4)	–	413 (42,8)	–	260 (25,8)	–	588 (15,8)	–	996 (25,2)
Einkommen (€)	–	–	–	–	–	–	–	–	3530	1244,2 ± 623,0	3804	1459,9 ± 759,8
*Rauchstatus*												
Nie-Raucher	650	265 (40,8)	1355	809 (59,7)	963	451 (46,8)	1037	545 (52,6)	3724	1317 (35,4)	3955	1426 (36,1)
Ex-Raucher	–	140 (21,5)	–	364 (26,9)	–	238 (24,7)	–	369 (35,6)	–	1258 (33,8)	–	1426 (36,1)
Raucher	–	245 (37,7)	–	182 (13,4)	–	274 (28,5)	–	123 (11,8)	–	1149 (30,8)	–	1103 (27,8)
*Region*												
Westdeutschland	655	449 (68,6)	1367	891 (65,2)	966	683 (70,7)	1042	713 (68,4)	–	–	–	–
Ostdeutschland	–	206 (31,4)	–	476 (34,8)	–	283 (29,3)	–	329 (31,6)	–	–	–	–
BMI (kg/m^2^)	–	–	–	–	–	–	–	–	3729	27,5 ± 4,8	3964	28,1 ± 5,3
Diabetes	–	–	1367	200 (14,6)	966	19 (2,0)	1042	169 (16,2)	3727	378 (10,1)	3961	462 (11,7)
HbA1c (%)	–	–	–	–	–	–	–	–	3719	5,4 ± 0,9	3962	5,4 ± 0,8
Sport im Sommer oder Winter	–	–	–	–	–	–	–	–	3723	1536 (41,3)	3953	2716 (68,7)
Elektrische Zahnbürste	655	93 (14,2)	1367	67 (4,9)	966	461 (47,7)	1042	348 (33,4)	–	–	3589	940 (26,2)
Interdentalhygiene	655	193 (29,5)	1367	107 (7,8)	966	599 (62,0)	1042	518 (49,7)	3739	1133 (30,3)	3971	962 (24,2)
Verwendung von Mundwasser	655	169 (25,8)	1367	528 (38,6)	966	337 (34,9)	1042	443 (42,5)	–	–	3589	1231 (34,3)
Zahnputzhäufigkeit ≥ 2-mal täglich	646	518 (80,2)	944	771 (81,7)	963	800 (83,1)	1038	874 (84,2)	3354	2812 (83,8)	3567	3037 (85,1)
Parodontosebehandlung^a^	646	165 (25,5)	–	–	956	192 (20,1)	1026	427 (41,6)	–	–	3568	692 (19,4)
*Kontrollbesuche beim Zahnarzt*^*b*^												
Vorsorge	653	451 (69,1)	1354	771 (56,9)	961	737 (76,7)	1041	951 (91,4)	–	–	–	–
Schmerzen/Probleme	–	202 (30,9)	–	583 (43,1)	–	224 (23,3)	–	90 (8,6)	–	–	–	–
Zahnarztbesuch innerhalb der letzten 12 Monate	653	565 (86,5)	1362	1012 (74,3)	960	834 (86,9)	1021	908 (88,9)	3715	3216 (86,6)	3158	3158 (100)
*Wie viel kann man selbst tun?*												
Nichts bis einiges	652	139 (21,3)	1356	395 (29,1)	963	139 (14,4)	1041	249 (23,9)	3705	616 (16,6)	3585	543 (15,2)
Viel	–	264 (40,5)	–	549 (40,5)	–	444 (46,1)	–	411 (39,5)	–	1694 (45,7)	–	1543 (43,0)
Sehr viel	–	249 (38,2)	–	412 (30,4)	–	380 (39,5)	–	381 (36,6)	–	1395 (37,7)	–	1499 (41,8)

Der Übersichtlichkeit halber wurde auf Angaben zu DMS IV verzichtet

*BMI* Body-Mass-Index, *HbA1c* Hämoglobin A1c, *N* Gesamtzahl

^a^DMS: jemals; SHIP: innerhalb der letzten 5 Jahre

^b^DMS: Beantwortung der Frage: „Gehen Sie zum Zahnarzt nur, wenn Sie Schmerzen oder Beschwerden haben? Oder gehen Sie regelmäßig oder manchmal auch zur Kontrolle?“

### Karies

**Figure 1:**
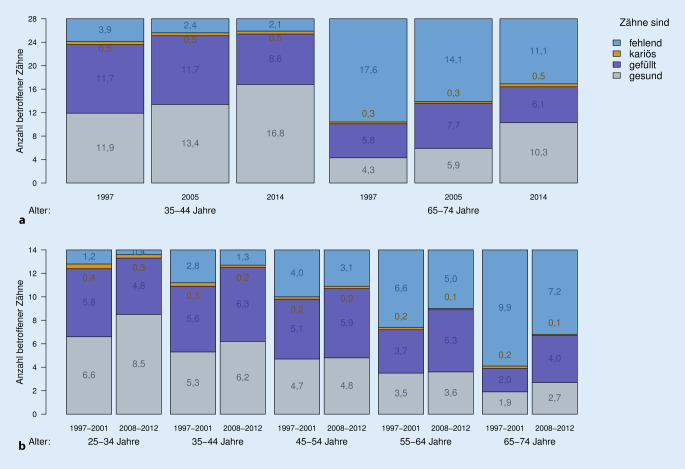

In DMS veränderte sich der Anteil der kariösen Zähne in beiden Altersgruppen über die Zeit nicht (Abb. 1 und Onlinematerial Tabelle Z1). Hingegen sank bei den 35- bis 44-Jährigen die Anzahl fehlender Zähne von 3,9 in DMS III auf 2,1 in DMS V und die Anzahl gesunder Zähne stieg von 11,9 auf 16,8. Bei den 65- bis 74-Jährigen sank die Anzahl fehlender Zähne von 17,6 auf 11,1 bzw. die Anzahl gesunder Zähne stieg von 4,3 auf 10,3 an. In SHIP zeichnete sich ein sehr ähnlicher, wenn auch abgeschwächter Trend ab. Bei den 35- bis 44-Jährigen sank der halbseitige Zahnverlust von 2,8 auf 1,3 und die Anzahl gesunder Zähne stieg von 5,3 auf 6,2. Bei den 65- bis 74-Jährigen verringerte sich halbseitig die Anzahl fehlender Zähne um 2,7, während die Anzahl der gesunden Zähne um 0,8 anstieg. (Um DMS grob mit SHIP zu vergleichen, können die SHIP-Zahlen einfach verdoppelt werden.)

### Parodontitis

**Figure 2:**
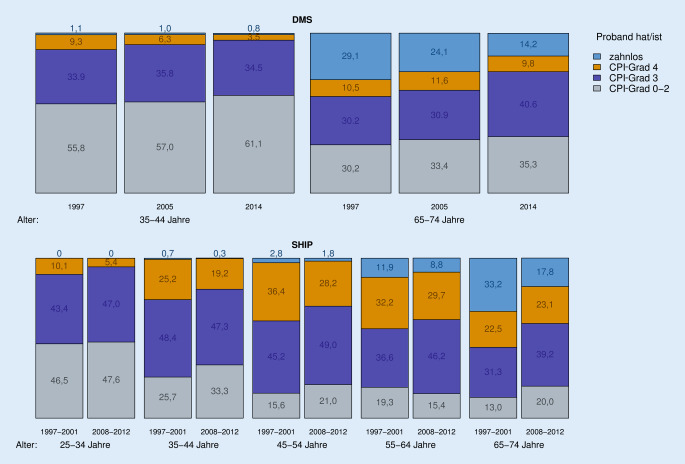

In der DMS fiel die Prävalenz des CPI-Grades 4 bei den 35- bis 44-Jährigen von 9,3 % auf 3,5 % und bei den Senioren wurde 2014 mit 9,8 % in etwa das Niveau von 1997 mit 10,5 % erreicht (Abb. 2 und Onlinematerial Tabelle Z2). Gleichzeitig hat sich der Anteil der zahnlosen 65- bis 74-Jährigen von 29,1 % auf 14,2 % halbiert. Im Gegenzug haben die Prävalenzen der CPI-Grade 0–2 und 3 kontinuierlich zugenommen. In SHIP war in allen Altersklassen eine Abnahme der Zahnlosen zu beobachten; bei den 65- bis 74-Jährigen halbierte sich der Anteil der Zahnlosen auf 17,8 %. Gleichzeitig zeichnete sich in den meisten Altersklassen eine Verschiebung vom CPI-Grad 4 hin zu CPI-Graden 0–3 ab.

**Figure 3:**
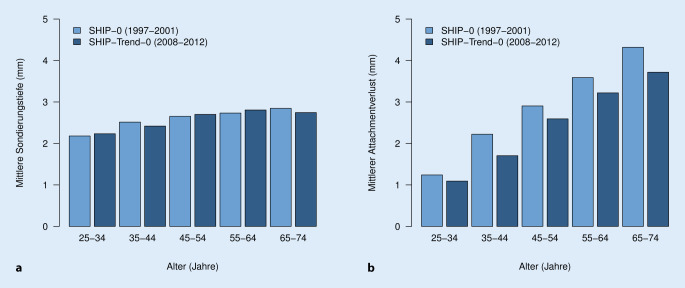

In Abb. 3 sind die Veränderungen der mittleren Sondierungstiefe und des mittleren Attachmentverlustes über die gesamte Altersspanne von SHIP‑0 zu SHIP-Trend‑0 aufgetragen. Es ist deutlich zu erkennen, dass die mittlere Sondierungstiefe von den jüngeren zu den älteren Probanden nur unwesentlich anstieg (von 2,3 mm auf 2,5 mm) und es kaum eine Veränderung zwischen SHIP‑0 und SHIP-Trend‑0 gab. Hingegen stieg der mittlere Attachmentverlust mit zunehmendem Alter linear von 1,3 mm auf 4,3 mm an. In allen Altersgruppen fielen die mittleren Attachmentverluste in SHIP-Trend‑0 um ca. 0,5 mm geringer aus als in SHIP‑0.

### Zahnverlust

In DMS stieg bei den 35- bis 44-Jährigen von 1997–2014 die Anzahl der Zähne von 23,8 auf 25,9 und bei den 65- bis 74-Jährigen von 10,4 Zähne auf 16,9 Zähne (Tab. 2). Dieser Trend konnte auch regional durch SHIP bestätigt werden. Bei den 65- bis 74-Jährigen der DMS verringerte sich Anteil der totalen Zahnlosigkeit von 24,8 % im Jahr 1997 auf 12,4 % im Jahr 2014. Entsprechend stieg die Zahnzahl bei bezahnten 65- bis 74-Jährigen von 13,9 auf 19,3 Zähne an. Auch in SHIP halbierte sich im Beobachtungszeitraum der Anteil der zahnlosen 65- bis 74-Jährigen von 32,7 % auf 15,7 %.

**Table 2:** 

	Anzahl *N*	Totale Zahnlosigkeit in %	Zahnzahl; alle Probanden	Zahnzahl; Bezahnte
**DMS**				
*35–44 Jahre*				
1997	655	1,1	23,8 ± 4,9	24,1 ± 4,2
2005	925	1,0	25,3 ± 4,0	25,5 ± 3,2
2014	966	0,8	25,9 ± 3,4	26,1 ± 2,6
*65–74 Jahre*				
1997	964	24,8	10,4 ± 9,0	13,9 ± 7,8
2005	1040	22,6	13,8 ± 9,8	17,8 ± 7,2
2014	1042	12,4	16,9 ± 9,1	19,3 ± 6,9
**SHIP**				
*25–34 Jahre*				
1997–2001	698	0	25,2 ± 2,9	25,2 ± 2,9
2008–2012	581	0	26,8 ± 1,8	26,8 ± 1,8
*35–44 Jahre*				
1997–2001	755	0,7	22,1 ± 4,9	22,2 ± 4,6
2008–2012	763	0,3	25,0 ± 4,0	25,1 ± 3,7
*45–54 Jahre*				
1997–2001	744	2,8	19,9 ± 7,4	20,5 ± 6,6
2008–2012	886	1,7	21,5 ± 6,2	21,8 ± 5,7
*55–64 Jahre*				
1997–2001	842	11,7	14,7 ± 9,1	16,7 ± 7,7
2008–2012	907	7,9	17,5 ± 9,5	19,0 ± 7,9
*65–74 Jahre*				
1997–2001	682	32,7	8,1 ± 9,5	12,1 ± 8,4
2008–2012	756	15,7	13,2 ± 9,6	15,7 ± 8,3

## Diskussion

### Zusammenfassung der wichtigsten Ergebnisse

Die in den letzten Jahrzehnten bei Kindern in Deutschland beobachtete massive Kariesabnahme (DMFT) von 8,8 kariösen, gefüllten oder fehlenden Zähnen (1983) auf 0,44 (2016; [15]) konnte auch bei den 35- bis 44- und den 65- bis 74-jährigen DMS- und SHIP-Teilnehmern, wenn auch in vermindertem Umfang, beobachtet werden. In den aktuellsten Studien DMS V und SHIP-Trend‑0 konnten im Vergleich zu den Vorgängerstudien DMS III bzw. SHIP‑0 bei den jüngeren Teilnehmern insgesamt mehr Zähne erhalten werden, vor allem mehr gesunde (füllungsfreie) Zähne. Hingegen stieg bei den älteren Teilnehmern in beiden Studien parallel zur Gesamtzahnzahl auch die Anzahl der gefüllten Zähne an. Nichtsdestotrotz konnten bei ihnen mehr gefüllte Zähne erhalten werden als noch in DMS III und SHIP‑0. Diese Beobachtungen passen mit den Abrechnungsdaten der gesetzlichen Krankenkassen zusammen: Zwischen 1997 und 2018 reduzierte sich die Anzahl der gelegten Füllungen von 67.914 auf 49.671 Mio. und die Anzahl der Extraktionen von 14.349 auf 12.417 Mio. [16]. Im längerfristigen Vergleich ist auch erkennbar, dass die Zahl der gelegten Füllungen noch deutlich stärker rückläufig war als die Zahl der Extraktionen.

### Veränderung der oralen Gesundheit

Der Sanierungsbedarf ist seit 1997 unverändert klein: Im Schnitt wiesen 0,3 bis 0,4 Zähne eine behandlungsbedürftige Kavität auf. Diese geringe Quote lässt sich sicherlich damit begründen, dass über 85 % der Bevölkerung mindestens einmal jährlich zum Zahnarzt gehen und weit über zwei Drittel der Patienten aus Kontrollgründen und nicht ausschließlich aufgrund von Schmerzen oder anderen Problemen zum Zahnarzt gehen. Die Halbierung der Zahnlosigkeit bei den 65- bis 74-Jährigen ist wahrscheinlich auf den massiven Kariesrückgang und eine stärkere Fokussierung der zahnärztlichen Behandlung auf den Zahnerhalt zurückzuführen [18]. Diese massive Abnahme der totalen Zahnlosigkeit stimmt mit einem weltweiten Trend überein [4].

Aus den DMS-Daten können keine belastbaren Rückschlüsse auf eine Veränderung der parodontalen Situation gezogen werden. Zum einen wurde das Untersuchungsprotokoll zwischen DMS III und DMS V geändert, sodass für den Vergleich der beiden Studien nur Daten von sehr wenigen Zähnen und Zahnflächen herangezogen werden konnten. Damit sind die Befunde nur bedingt belastbar und führen zu einer Unterschätzung der tatsächlichen Prävalenzen in den DMS. Zum anderen wurden ausschließlich Sondierungstiefen gemessen. Sondierungstiefen spiegeln die aktuelle Erkrankungslast wider, da der Zahnarzt durch eine effektive Parodontalbehandlung nur die Sondierungstiefen reduzieren kann. Er hat jedoch kaum Einfluss auf die Veränderung der Attachmentverluste. Letztlich sei angemerkt, dass ein Vergleich der CPI-Werte aus DMS und SHIP aufgrund der unterschiedlichen Teilbefundprotokolle nicht möglich ist.

Aus den SHIP-Daten ist hingegen klar zu erkennen, dass die mittleren Attachmentverluste sich verbessert haben, während sich die mittleren Sondierungstiefen nur unwesentlich verändert haben [19]. Für den Versorgungsalltag bedeutet dies, dass die parodontale Behandlungslast im Beobachtungszeitraum nicht abgenommen, sondern im Gegenteil durch die Zunahme der Zahnzahl und die demografische Verschiebung hin zu einer älteren Bevölkerung wahrscheinlich zugenommen hat [20] und möglicherweise weiterhin zunehmen wird. Wahrscheinlich hat die in der SHIP-Population beobachtete Verminderung der mittleren Attachmentverluste auch in DMS stattgefunden. Sollte dem so sein, kann vermutet werden, dass die verminderten Attachmentverluste zu weniger Extraktionen und damit auch zu einem größeren Zahnerhalt beigetragen haben. Dies ist damit zu begründen, dass ein Zahnarzt sich aufgrund der Zahnlockerung beziehungsweise des Knochenabbaus zu einer Extraktion aus parodontalen Gründen entscheidet [21].

### Ursachen für die Verbesserung der Mundgesundheit

Zum einen hängen die beobachteten Trends mit der Verbesserung der Mundgesundheit und der grundlegenden Verschiebung von einer restaurativen zu einer präventiven Zahnmedizin zusammen. Der Rückgang in der Füllungstherapie liegt vermutlich, wie bei den Kindern, hauptsächlich in der Einführung der fluoridierten Zahnpasta in den 1970er-Jahren begründet [17]. Der Rückgang der Extraktionen könnte, außer durch die abnehmende Karieslast, auch durch die Absenkung des Zuschusses für prothetische Leistungen 1997/1998 und die Einführung des Festkostenzuschusses für prothetische Versorgungen 2004/2005 beeinflusst worden sein. Wahrscheinlich ist die Halbierung der Zahnlosigkeit bei den 65- bis 74-Jährigen auf dieselben Gründe zurückzuführen. Aus unseren Analysen kann nicht abgeleitet werden, ob die Individualprophylaxe einen Einfluss auf den Zahnerhalt hat, da dazu in DMS III und SHIP‑0 keine Angaben vorlagen.

Zum anderen ist eine Betrachtung der Veränderung der bekannten Risikofaktoren unumgänglich, wenn man verstehen möchte, warum sich die Prävalenz der Karies und der Parodontitis und somit auch die Zahnverlustraten in den letzten Jahren verändert haben. Die wesentlichen Risikofaktoren umfassen den sozioökonomischen Status, welcher sich wiederum auf die Prävalenzen von Rauchen, Diabetes mellitus, Übergewicht und mangelnder Bewegung sowie auf die Mundhygiene auswirkt. Die Relevanz einiger dieser Faktoren wird anschließend kurz diskutiert.

Der sozioökonomische Status wird in DMS und SHIP anhand der Schulbildung erhoben. Als möglicher kausaler Pfad zwischen Schulbildung und Gesundheit werden bessere Chancen auf dem Arbeitsmarkt, ein höheres Einkommen, bessere kognitive und emotionale Fähigkeiten und größere soziale Netzwerke angesehen [22–24]. Zum Beispiel können Personen mit höherem Bildungstand und Einkommen mehr Geld für häusliche und professionelle orale Prophylaxe ausgeben. Darüber hinaus ist Rauchen oder übermäßiger Konsum von hochkalorischer Nahrung bei Personen mit kürzerem Schulbesuch häufiger zu beobachten [25]. In einer Auswertung von englischen Kohorten konnte gezeigt werden, dass durch einen um ein Jahr verlängerten Schulbesuch die Wahrscheinlichkeit von Zahnlosigkeit um 9 % vermindert wurde [26]. Nach der Theorie des „Humankapitals“ kann Bildung somit als Investition in die künftige Gesundheit betrachtet werden [27].

Die Raucherprävalenz hat sich in beiden Studien nicht sehr stark verändert. Diese geringfügige Abnahme stimmt mit den deutschlandweiten Untersuchungen des Robert Koch-Instituts überein. So sank bei Männern im Alter zwischen 25 und 69 Jahren die Raucherprävalenz von 37,6 % (1998) auf 34,9 % (2012; [28]). Rauchen wirkt sich vor allem auf die parodontale und nicht auf die Kariessituation aus und kann langfristig den parodontal induzierten Zahnverlust beeinflussen [29]. In einem Review schätzte Bergström, dass Rauchen das Risiko für Parodontitis um das 5‑ bis 20-Fache erhöht [30]. Er konnte auch zeigen, dass eine abnehmende Raucherprävalenz mit einer Abnahme der Parodontitis in der Bevölkerung einhergeht [31].

Neben der Schulbildung hatte möglicherweise die häuslich durchgeführte Mundhygiene einen wesentlichen Einfluss auf den Erhalt zusätzlicher Zähne in DMS und SHIP. Die Anwendung von Hilfsmitteln zur Interdentalhygiene hat laut DMS in den letzten 17 Jahren um 33 % bei den 35- bis 44-Jährigen und um 44 % bei den 65- bis 74-Jährigen zugenommen. Neuere Metaanalysen konnten für interdentale Hilfsmittel jedoch nur eine mäßige Evidenz für die Reduktion von Plaque und Zahnfleischentzündung (Gingivitis) und keine Evidenz für die Reduktion patientenrelevanter Endpunkte wie Approximalkaries (Interdentalkaries), Parodontitis oder Zahnverlust nachweisen [32, 33]. Hingegen lieferte eine vertiefte epidemiologische Analyse der DMS-Daten Hinweise, dass interdentale Hilfsmittel neben den fluoridierten Zahnpasten ebenso, wenn auch nur mäßig, zur Kariesprävention beitrugen und dadurch mehr Zähne erhalten wurden [34]. Diese Ergebnisse stehen im Einklang mit einer kürzlich veröffentlichten Studie, in der durch die Anwendung von Zahnseide das Ausmaß von Parodontitis, Karies und Zahnverlust über 5 Jahre bei Senioren (> 65 Jahre) verringert wurde [35]. Außerdem stieg von DMS III zu DMS V die Anwendung von elektrischen Zahnbürsten von 14,9 % auf 48,3 % bei den 35- bis 44-Jährigen und von 7,2 % auf 36,9 % bei den 65- bis 74-Jährigen. Laut einer 11-jährigen longitudinalen Untersuchung, bei der Anwender einer elektrischen Zahnbürste eine verringerte Parodontitisprogression und eine erhöhte Zahnretention aufwiesen [36], kann man davon ausgehen, dass der Gebrauch elektrischer Zahnbürsten nicht nur dem manuell eingeschränkten Benutzer, sondern auch dem normalen Konsumenten einen Vorteil bringt. Zusammengefasst bedeutet dies, dass die Industrie und die Zahnärzte mit ihren Teams eine hervorragende deutschlandweite Aufklärungsarbeit betrieben haben, die in den letzten 17 Jahren zu einer deutlichen Verbesserung der oralen Gesundheit durch eine verbesserte Mundhygiene geführt hat.

### Mögliche Präventionsstrategien

Die Ergebnisse unserer Analysen illustrieren hervorragend die von Rose benannten Strategien des ärztlichen Handelns: die Hochrisikostrategie („high-risk strategy“) sowie die Bevölkerungsstrategie („population strategy“; [37]). Unter dem Begriff der „Hochrisikostrategie“ versteht man das Fokussieren der Zahnärzte auf individuelle Patienten mit einer großen Krankheitslast, z. B. manifester Karies. Hier konnten sie durch Füllungen ein weiteres Fortschreiten der Karies verhindern (wie die kleine Anzahl unversorgter kariöser Läsionen zeigt) und schlussendlich eine Wurzelbehandlung unnötig machen. Dies spiegelt sich für den Beobachtungszeitraum der DMS auch in der Abnahme der abgerechneten Füllungen sowie in der zeitverzögert auftretenden Abnahme der abgerechneten Wurzelfüllungen wider [18]. Dieser Strategie gegenüber stellt Rose die „Bevölkerungsstrategie“, die dem Individuum nur einen kleinen, eventuell unmerklichen gesundheitlichen Nutzen, der Bevölkerung insgesamt aber einen großen Vorteil bringt. Die vermehrte Anwendung von interdentalen Hilfsmitteln und der elektrischen Zahnbürste illustrieren diesen Aspekt hervorragend. Obwohl auf individueller Patientenebene anhand von Metaanalysen für interdentale Hilfsmittel und elektrische Zahnbürsten keine patientenrelevanten Verbesserungen nachgewiesen werden konnten, wurde auf Populationsebene tatsächlich ein sehr großer Nutzen nachgewiesen [38–40]. Vermutlich hat die Anwendung fluoridierter Zahnpasten einen noch größeren Nutzen für die Bevölkerung. Immerhin sind inzwischen über 90 % der Zahnpasten in Deutschland fluoridiert. In DMS und SHIP liegen allerdings keine Daten zur Fluoridierung von Zahnpasten vor, sodass deren Einfluss auf die Kariesgesundheit nicht analysiert werden konnte.

Die in der ehemaligen Deutschen Demokratischen Republik (DDR) insbesondere durch die Kinderstomatologie durchgeführte staatlich organisierte Gesundheitsfürsorge hatte bei den 12-Jährigen anscheinend guten Erfolg: Die nach der Wende (1992) durchgeführte DMS II konnte zeigen, dass Kinder in Ostdeutschland im Durchschnitt fast einen Zahn weniger mit Karieserfahrung aufwiesen [41]. Ebenso waren mehr 12-Jährige kariesfrei als in Westdeutschland. Dies könnte evtl. auch mit dem verbreiteten Einsatz von Fluoridtabletten und mit der Trinkwasserfluoridierung erklärt werden. Mit der DMS II konnte außerdem gezeigt werden, dass die jüngeren Erwachsenen (35- bis 44-Jährige) in Ostdeutschland 3 kariesfreie Zähne mehr hatten als jene in Westdeutschland [41]. Andererseits bestanden erhebliche Unterschiede in der prothetischen Versorgung. In Westdeutschland war der Anteil prothetisch ersetzter Zähne um 22 Prozentpunkte höher als in Ostdeutschland. Zusätzlich fehlte bei den 35- bis 44-Jährigen in Ostdeutschland durchschnittlich bereits ein Zahn mehr als bei denen in Westdeutschland. Mit der Wiedervereinigung ist es in den „neuen Bundesländern“ zu tiefgreifenden Veränderungen der wirtschaftlichen und gesundheitspolitischen Situation gekommen, aber auch auf die „alten Bundesländer“ traf dies zum Teil zu. Mit der Einführung der Individual- und Gruppenprophylaxe für Kinder und Jugendliche sowie durch die breitere Verfügbarkeit von Fluoriden in den Zahnpasten gingen die Karieserkrankungen zurück: So wurde in der DMS III (1997) sowohl ein Rückgang der Karieserfahrung als auch eine Zunahme kariesfreier Gebisse verzeichnet, und zwar gleichermaßen in West- wie in Ostdeutschland [11]. Dennoch zeigten die DMS-III-Ergebnisse, dass eine Angleichung der Verhältnisse in West- und Ostdeutschland noch nicht stattgefunden hatte – vielmehr wurde dieser Trend erst mit der DMS IV deutlich [10]. Inzwischen ist für die Mundgesundheit jedoch eine nachhaltige Entwicklung hin zu einer West-Ost-Angleichung erkennbar [8, 10]. Bei Kindern gibt es nur noch geringfügige Unterschiede hinsichtlich Kariesfreiheit und Karieserfahrung. Eine deutliche Angleichung sowohl der Karieserfahrung als auch der Anzahl fehlender Zähne wurde auch bei den Erwachsenen beobachtet. Ebenso hat sich der Anteil zahnprothetischer Versorgungen deutlich angeglichen.

### Stärken und Schwächen

Die Stärken von DMS und SHIP umfassen den populationsbasierten Ansatz, den großen Stichprobenumfang, eine umfassende Schulung und Zertifizierung der Untersucher sowie eine qualitativ hochwertige Datenerhebung. Als Schwäche sollte die unterschiedliche Response der Studien diskutiert werden. Während in DMS III, DMS IV und SHIP‑0 noch über 60 % der ausgewählten Personen an der Studie teilnahmen, waren es in DMS V und SHIP-Trend‑0 nur noch 50 %. Eine ähnliche Abnahme der Teilnahmebereitschaft wurde auch bei anderen Bevölkerungsstudien beobachtet [42–44]. Um einer Verzerrung der Ergebnisse entgegenzuwirken, wurden die Daten in beiden Studien gewichtet (für DMS nach Geschlecht, Altersjahrgang, Bundesland, BIK-Gemeindegrößenklasse und Schulausbildung; für SHIP nach Alter, Geschlecht und Methode der Stichprobenziehung). Falls dennoch Verzerrungen bei den Prävalenzschätzern aufgetreten sein könnten, wären deren Auswirkungen auf die Veränderung der Erkrankungsprävalenzen nur schwer abzuschätzen. Als weitere Schwäche ist die Teilbefundung beim Parodontalbefund aufzuführen [45, 46], wodurch eine Bewertung der aktuellen parodontalen Situation nur eingeschränkt möglich ist. Allerdings kann davon ausgegangen werden, dass die Teilbefundung gleichermaßen für die einzelnen Erhebungen in SHIP als auch in den DMS zu einer Unterschätzung der CPI-Prävalenzen geführt hat, wodurch keine übermäßigen Verzerrungen im Hinblick auf den Trend der CPI-Grade 3–4 zu erwarten sind. Weiterhin ist anzumerken, dass für SHIP auch der Kariesbefund nur halbseitig erhoben wurde. Unter der Annahme, dass die Verteilung der Karies symmetrisch ist [47], kann von geringfügigen Verzerrungen der DMFT-Werte ausgegangen werden. Letztlich sei anzumerken, dass die klinische Befundung allein die Prävalenz der proximalen Karies im Vergleich zur Kombination von visueller Inspektion und Röntgen unterschätzt [48].

## Fazit

Die bevölkerungsbasierten Deutschen Mundgesundheitsstudien (DMS) und Studies of Health in Pomerania (SHIP) zeigten konsistent eine Zunahme gesunder, füllungsfreier Zähne bei gleichbleibend geringen Prävalenzen kariöser Läsionen, eine geringfügige Reduktion der Probanden mit einer schweren parodontalen Erkrankung sowie über alle Altersklassen hinweg mehr Zahnerhalt und weniger Zahnlosigkeit, insbesondere bei den über 65-Jährigen. Für die Zukunft ist eine Fortführung dieser Trends zu erwarten. Bedingt durch den höheren Zahnerhalt und die aktuellen demografischen Entwicklungen hin zu einer alternden Bevölkerung ist auf Populationsebene in Zukunft allerdings eher mit einer erhöhten parodontalen Behandlungsbedürftigkeit zu rechnen. Zur Verbesserung der Zahn- und Mundgesundheit haben sich bisher verschiedene Präventionsstrategien, die sowohl auf Populationsebene als auch auf individueller Ebene greifen, als sinnvoll herausgestellt. Für die Zukunft erscheint es daher wichtig, auf der einen Seite Präventionsstrategien zielgerichtet umzusetzen und auf der anderen Seite den möglicherweise erhöhten parodontalen Behandlungsbedarf abzudecken.

## Supplementary Information


