# Supplementary material for: Trends in dental and oral health status in Germany between 1997 and 2014
Source: Bundesgesundheitsblatt Gesundheitsforschung Gesundheitsschutz. 2021 Jun 18;64(7):782–92. [Article in German] doi: 10.1007/s00103-021-03345-6 (PMC8241795; doi:10.1007/s00103-021-03345-6)
Supplement: Supplementary file 1 [file 103_2021_3345_MOESM1_ESM.pdf]

Bundesgesundheitsblatt 7/2021

Elektronisches Zusatzmaterial zum Beitrag:

Entwicklung der Zahn- und Mundgesundheit in Deutschland von 1997 bis 2014

Thomas Kocher[1], Birte Holtfreter[1], Vinay Pitchika[1], Kathrin Kuhr[2], Rainer A. Jordan[2]

[1] Poliklinik für Zahnerhaltung, Parodontologie, Endodontologie, Kinderzahnheilkunde und Präventive Zahnheilkunde, Universitätsmedizin Greifswald, Greifswald, Deutschland
[2] Institut der Deutschen Zahnärzte (IDZ), Köln, Deutschland

Korrespondenzadresse
Prof. Dr. Thomas Kocher
Poliklinik für Zahnerhaltung, Parodontologie, Endodontologie, Kinderzahnheilkunde und Präventive Zahnheilkunde, Universitätsmedizin Greifswald
Fleischmannstraße 42
17475 Greifswald
Deutschland
kocher@uni-greifswald.de

Inhalt:

Ergänzung zur Methode: Erhebung des Parodontalstatus

Tabelle Z1: Verteilung des DMF-T und seiner Einzelkomponenten in den Deutschen Mundgesundheitsstudien (DMS) und der Study of Health in Pomerania (SHIP).

Tabelle Z2: Verteilung des Community Periodontal Indexes (CPI) unter Berücksichtigung der Zahnlosigkeit in den Deutschen Mundgesundheitsstudien (DMS) und der Study of Health in Pomerania (SHIP).

Ergänzung zur Methode: Erhebung des Parodontalstatus

Die parodontale Befundung erfolgte mit Hilfe einer manuellen Parodontalsonde (SHIP-0: PCP11; SHIP-Trend-0: PCP15; DMS: PCP 11.5B; Hu-Friedy, Tuttlingen, Deutschland). In SHIP wurde die Sondierungstiefe halbseitig (1./4. oder 2./3. Quadrant; in SHIP-0 alternierend, in SHIP-Trend-0 randomisiert ausgewählt) an vier Stellen pro Zahn (mesiobukkal, mittbukkal, distobukkal, mittoral) erfasst. In DMS III wurde die Sondierungstiefe halbseitig (1./4. Quadrant) an zwei Stellen (mesiobukkal und mittbukkal) erfasst, während in DMS IV und V jeweils drei Stellen (mesiobukkal, mittbukkal, distooral) aller 12 Indexzähne (17, 16, 11, 24, 26, 27, 37, 36, 31, 44, 46, 47) befundet wurden.

Tabelle Z1: Verteilung des DMF-T und seiner Einzelkomponenten in den Deutschen Mundgesundheitsstudien (DMS) und der Study of Health in Pomerania (SHIP) nach Altersgruppen. Angabe von Mittelwerten für die Anzahl der jeweils betroffenen Zähne.

	N	Anzahl gesunder Zähne (ST)	Anzahl gefüllter Zähne (FT)	Anzahl kariöser Zähne (DT)	Anzahl fehlender Zähne (MT)	Anzahl kariöser, gefüllter oder fehlender Zähne (DMFT)
DMS						
35-44 Jahre						
1997	655	11,9	11,7	0,5	3,9	16,1
2005	925	13,4	11,7	0,5	2,4	14,5
2014	966	16,8	8,6	0,5	2,1	11,2
65-74 Jahre						
1997	1367	4,3	5,8	0,3	17,6	23,6
2005	1040	5,9	7,7	0,3	14,1	22,1
2014	1042	10,3	6,1	0,5	11,1	17,7
SHIP (halbseitig)						
25-34 Jahre						
1997-2001	698	6,6	5,8	0,4	1,2	7,4
2008-2012	534	8,5	4,8	0,3	0,4	5,5
35-44 Jahre						
1997-2001	755	5,3	5,6	0,3	2,8	8,7
2008-2012	714	6,2	6,3	0,2	1,3	7,8
45-54 Jahre						
1997-2001	744	4,7	5,1	0,2	4,0	9,3
2008-2012	833	4,8	5,9	0,2	3,1	9,2
55-64 Jahre						
1997-2001	842	3,5	3,7	0,2	6,6	10,5
2008-2012	826	3,6	5,3	0,1	5,0	10,4
65-74 Jahre						
1997-2001	681	1,9	2,0	0,2	9,9	12,1
2008-2012	659	2,7	4,0	0,1	7,2	11,3

Berechnung unter Berücksichtigung der zahnlosen Probanden. SHIP: Befunderhebung halbseitig (maximal 14 Zähne, 64 Flächen). Abkürzungen: N, Anzahl; DMFT, Decayed, Missing, Filled Teeth; DT, Decayed Teeth; FT, Filled Teeth; MT, Missing Teeth; ST, Sound Teeth.

Tabelle Z2: Verteilung des Community Periodontal Indexes (CPI) unter Berücksichtigung der Zahnlosigkeit in den Deutschen Mundgesundheitsstudien (DMS) und den Studies of Health in Pomerania (SHIP) nach Altersgruppen. Darstellung relativer Häufigkeiten für die einzelnen CPI Grade sowie Zahnlosigkeit.

	N	CPI-Grade 0-2	CPI-Grad 3	CPI-Grad 4	Zahnlos
DMS (Zähne 17, 16, 11, 44, 46 und 47; 2 Flächen)					
35-44 Jahre					
1997	648	55,8%	33,9%	9,3%	1,1%
2005	920	57,0%	35,8%	6,3%	1,0%
2014	884	61,1%	34,5%	3,5%	0,8%
65-74 Jahre					
1997	1169	30,2%	30,2%	10,5%	29,1%
2005	977	33,4%	30,9%	11,6%	24,1%
2014	914	35,3%	40,6%	9,8%	14,2%
SHIP (halbseitig; 4 Flächen)					
25-34 Jahre					
1997-2001	693	46,5%	43,4%	10,1%	0%
2008-2012	531	47,6%	47,0%	5,4%	0%
35-44 Jahre					
1997-2001	749	25,7%	48,4%	25,2%	0,7%
2008-2012	707	33,3%	47,3%	19,2%	0,3%
45-54 Jahre					
1997-2001	741	15,6%	45,2%	36,4%	2,8%
2008-2012	818	21,0%	49,0%	28,2%	1,8%
55-64 Jahre					
1997-2001	829	19,3%	36,6%	32,2%	11,9%
2008-2012	815	15,4%	46,2%	29,7%	8,8%
65-74 Jahre					
1997-2001	664	13,0%	31,3%	22,5%	33,2%
2008-2012	664	20,0%	39,2%	23,1%	17,8%

Abkürzungen: N, Anzahl
